# Flexible and Tunable 3D Gold Nanocups Platform as Plasmonic Biosensor for Specific Dual LSPR-SERS Immuno-Detection

**DOI:** 10.1038/s41598-017-14694-1

**Published:** 2017-10-27

**Authors:** M. Focsan, A. M. Craciun, M. Potara, C. Leordean, A. Vulpoi, D. Maniu, S. Astilean

**Affiliations:** 1 Nanobiophotonics and Laser Microspectroscopy Center, Interdisciplinary Research Institute on Bio-Nano-Sciences, Babes-Bolyai University, Treboniu Laurean Str. 42, Cluj-Napoca, 400271 Romania; 2 Nanostructured Materials and Bio-Nano-Interfaces Center, Interdisciplinary Research Institute on Bio-Nano-Sciences, Babes-Bolyai University, Treboniu Laurian Str. 42, Cluj-Napoca, 400271 Romania; 3 Biomolecular Physics Department, Faculty of Physics, Babes-Bolyai University, M Kogalniceanu, Str. 1, Cluj-Napoca, 400084 Romania

## Abstract

Early medical diagnostic in nanomedicine requires the implementation of innovative nanosensors with highly sensitive, selective, and reliable biomarker detection abilities. In this paper, a dual Localized Surface Plasmon Resonance - Surface Enhanced Raman Scattering (LSPR- SERS) immunosensor based on a flexible three-dimensional (3D) gold (Au) nanocups platform has been implemented for the first time to operate as a relevant “proof-of-concept” for the specific detection of antigen-antibody binding events, using the human IgG - anti-human IgG recognition interaction as a model. Specifically, polydimethylsilane (PDMS) elastomer mold coated with a thin Au film employed for pattern replication of hexagonally close-packed monolayer of polystyrene nanospheres configuration has been employed as plasmonic nanoplatform to convey both SERS and LSPR readout signals, exhibiting both well-defined LSPR response and enhanced 3D electromagnetic field. Synergistic LSPR and SERS sensing use the same reproducible and large-area plasmonic nanoplatform providing complimentary information not only on the presence of anti-human IgG (by LSPR) but also to identify its specific molecular signature by SERS. The development of such smart flexible healthcare nanosensor platforms holds promise for mass production, opening thereby the doors for the next generation of portable point-of-care devices.

## Introduction

One of the top research priorities in nanomedicine is the implementation of nanotechnology-based diagnostics tools able to identify disease as early as possible, ideally at the level of a single molecule biomarker^1,2^. Early detection of specific disease biomarkers and effective diagnosis are important for disease screening, preventing epidemics and enabling physicians to provide the right therapy^3^. Specifically, a large number of disease biomarkers are proteins and their presence in biological fluids are considered an indicator of the presence of some diseases such as diabetes, cancers and so on^4^. Today, most of currently employed diagnostic procedures are time-consuming, costly, complex and invasive processes which imply sophisticated assays, including multi-step protocols and difficult fluid handling. Therefore, in the field of clinical diagnostic remains an urgent need to develop simple, affordable and accurate point-of-care (POC) procedures, particularly in resource constrained settings, to allow both rapid and portable determination of clinical protein biomarkers, where complex assays for protein analytes, such as enzyme-linked immunosorbent assay (ELISA), radio-immunoassay, Western blot or mass spectrometry, cannot be performed^5,6^.

In line with the above-mentioned requirements, the development of inexpensive and user-friendly diagnostic sensors for the detection of various biotargets with a very high sensitivity, selectivity and reliability represents an urgent and critical step toward the implementation of smart and early diagnostic procedures. To address this, significant research has been devoted in the last decade to fabricate various optical biosensors based on plasmonic transducers toward POC testing of different biomarkers present in blood, including Localized Surface Plasmon Resonance (LSPR), Surface-Enhanced Raman Scattering (SERS) or fluorescent devices^7–9^. In particular, the demand for LSPR sensing has lately increased owing to its label-free, portability, real-time and minimal interference performance. Compared to a conventional SPR sensor that is based on the excitation of propagating surface plasmons, so-called surface plasmon polaritons (SPPs), which are directly generated on a flat noble metallic thin surface (10–200 nm in thickness) using the Kretschmann-Raether prism geometry, LSPR -known as non-propagating surface plasmons- are generated on the surface of individual metallic nanoparticles of 10–200 nm in size^10^. The resonance wavelength of LSPR is strongly dependent on the nanoparticles’s type, size, shape, interparticle spacing, as well as the dielectric environment^11^. When a biomaterial is immobilized at the surface of the metal, any change due to mass accumulation is accompanied by a refractive index change which can be directly monitored by the SPP or LSPR spectral response. As a result, SPP-based biosensors are now considered as a leading technology for real-time detection and studies of biological binding events^12^. In particular, in the case of diagnostic applications, the LSPR detection process is triggered by the molecular recognition of the target biomarkers by detecting a measurable wavelength red-shift of the plasmonic band caused by the changes in the local refractive index around the metallic surface^13^. In this context, LSPR-based biosensors become a real alternative to the currently available tests on the market (e.g. standard SPR, Immunofluorescence or ELISA assays). As an example, the picomolar (pM) detection of various proteins like immunoglobulins, C-reactive protein and fibrinogen has been achieved using LPSR-based biosensors^14^. Moreover, our team has recently proposed a new strategy to improve the sensitivity of LSPR-based biosensor using the biotin-streptavidin recognition interaction as a proof-of-concept^15^. Specifically, the innovation of this biosensor consists in enhancing the LSPR response by using streptavidin-conjugated anisotropic Au nanorods compared to free streptavidin, the plasmonic nanobiosensors being employed herein as amplification labels. The enhancement of the wavelength shift by up to 400% recorded from a LSPR-based sensor was also obtained by Hall *et al*., labelling the antibody with spherical Au nanoparticles in order to achieve a more sensitive detection^16^. Later, the same group has described for the first time an LSPR imaging strategy created to perform multiple biosensing experiments simultaneously using a single large area array^17^. Despite high sensitivity in detecting the amount of biomaterial attached at the surface, the structure and molecular identification of immobilized targeted analyte is elusive in both SPP- and LSPR-based sensors. In this context, it becomes primordial to combine the LSPR investigation with other highly specific detection technique in order to obtain an unambiguous identification of the target analyte to achieve high-throughput analysis.

On the other hand, the excitation of LSPR enhances the local electromagnetic field promoting SERS. Significantly, ultrasensitive and specific biomarker detection by SERS could have several distinct advantages for direct POC applications considering the ability of Raman spectroscopy -as a powerful fingerprinting tool- to identify the target analytes at very low concentrations from complex chemical environment in a non-destructive manner, distinguishing simultaneously the molecular specific signature of each biomarker of interest by Raman intensity or frequency^18–20^. In particular, SERS detection is based on registering the vibrational Raman spectrum of the analyte via the amplification of the local electromagnetic field generated through the LSPR excitation of the nanostructured metallic surface. To note that in SERS, the total enhancement factor, which could increase from 10^3^ reaching 10^10^, arises from a combination of electromagnetic and chemical mechanisms^21,22^.

Periodically ordered plasmonic arrays can act as attractive and innovative types of plasmonic biosensors owing to their multiple advantages such as i) low-cost and large area fabrication with properly controlled sizes, which consequently implies high surface area for the antibodies attachment, providing thus greater specificity and sensitivity, ii) better structural controllability and uniformity, iii) strong three-dimensional (3D) local field amplification; iv) easy integration with microfluidics for label-free real time biomarkers detection and so on^23,24^. In this periodically ordered plasmonic arrays, localized and propagating SPR modes are intercoupled^25^, the resulting resonance mode depends on the material composition used in the sensor and its geometrical parameters, like periodicity, size, shape in the array. For example, our group has already succeeded in fabricating for the first time a multifunctional sensing platform for the detection of para-aminothiophenol (p-ATP) molecules by integrating LSPR transducers with SERS activity on low-cost subwavelength metallic ordered nanohole arrays^26^.

More importantly, the flexible platform-based technologies have recently created an exciting avenue in the field of portable POC diagnostic devices owing to their physiochemical properties as well as integrability into miniaturized and multiplexed analytical chips^27^. Kahraman *et al*. have firstly developed a simple method to fabricate a mechanically flexible plasmonic nanostructure based on the combination of soft lithography and thin silver film deposition by employing polydimethylsilane (PDMS) as elastomer and regular arrays of self-assembled nanosheres as mold by using spherical sulfate latex particles^28^. The as-obtained silver-coated flexible nanovoids have been used as efficient SERS platforms for the detection of a small Raman analyte (herein 4-ATP analyte). Later, these flexible silver nanostructures have been implemented by the same group for the label-free SERS detection of different proteins at low concentrations^29^ or for the capture of intact and ruptured exosomes in solution^30^, demonstrating thus the ability of these types of flexible nanoplatforms to be used for SERS-sensing applications. However, despite all these proved SERS-sensing abilities, there are no article in literature that made use of simultaneously integration of LSPR and SERS sensing on the same large-area flexible ordered plasmonic array nanoplatform in order to traduce antigen-antibody binding events that result from different immunological reactions.

Compared with the rigid substrates, such as glass, silicon, etc, flexible-based substrates are highly desirable for daily-life applications due to the possibility of using them onto irregular detection surfaces. However, to transform SERS into a real-world sensing detection platform, a lot of effort should be dedicated to optimize the capture and attachment of target biomarker from a complex environment not directly to the metal surface but to receptor molecules which are immobilized on the detection surface, giving specificity to the biosensor. The correct distance between target biomarkers and metal surface should be optimized to keep the detection in the enhanced region, the so-called “hot-spots”. Furthermore, the fabrication of a dual flexible LSPR-SERS plasmonic biosensor that could be successfully implemented for specific biomarker detection and can be further translated to real-world diagnostic applications still remains a challenge that should be addressed in literature. Therefore, there is a strong current scientific priority to efficiently implement both LSPR and SERS sensing onto the same reproducible and large area plasmonic nanoplatform in order to detect not only the presence of analytes/biomarkers (by LSPR) but also to unambiguously identify even multiple target analytes/biomarkers (by SERS), being thus closer to a real molecular diagnostic application.

In this study, we demonstrate the feasibility of a large-scale and mechanically flexible 3D Au nanocups-based platform to be used as active dual biosensor for the detection of the specific human IgG - anti-human IgG recognition interaction as a “proof-of-concept” *via* both LSPR and SERS. Specifically, the flexible Au platform has been designed using an adapted approach previously developed by Wachsmann-Hogiu’s group^28,30^ that use a thin plasmonic film coated polydimethylsilane (PDMS) elastomer mold employed for pattern replication of hexagonally close-packed monolayer of the polystyrene nanospheres (PS) configuration. One of the advantages of this fabrication approach is the possibility to obtain highly reproducible and tunable large-area nanocups array by simply changing the size of PS used herein as templates (*i.e*. PS with diameters of 527 nm (PS 527), 600 nm (PS 600), 719 nm (PS 719) and PS 836 (PS 836), respectively), which consequently will generate tunable plasmonic responses. Additionally, and importantly, the Au film deposited on the flexible nanocups as plasmonic coating material provides excellent biocompatibility of the designed nanoplatform^31^. The plasmonic tunability as well as the confined plasmonic field of the as-fabricated Au nanoplatform were theoretically evaluated by Finite-Difference Time Domain (FDTD) simulations and compared with experimental optical responses and SERS performance using 4-mercaptobenzoic acid (4-MBA) as a probe molecule. Subsequently, we have purposely performed our “proof-of-concept” demonstration for specific human IgG - anti-human IgG recognition interaction, employed as a model, with the nanoplatform having the highest SERS activity. In particular, the successive red-shifts of the optical response of the nanoplatform associated with the increased local refractive index reveal the immobilization of human IgG onto the chemically-modified flexible Au nanocups and the specific capture of the target anti-human IgG by human IgG-functionalized nanoplatform. But, considering that the specific molecular identification of the recognition interaction still remains elusive by recording plasmonic response alone, we have complemented our detection with reliable SERS measurements, which prove the capture of human-IgG, certifying thus this antigen-antibody binding event. Until now, in our knowledge, SERS has not been implemented in combination with LSPR-based sensing to traduce antigen-antibody binding events in immuno-analysis employing a flexible Au nanocups array platform. As such, by implementing a synergistic LSPR/SERS-based sensing on the same reproducible and large-area flexible Au nanoplatform we are able provide complimentary information not only on the presence of anti-human IgG (by LSPR investigation) but also to identify its specific molecular signature by SERS, being closer to the next generation of smart flexible healthcare nanosensor platforms.

## Results and Discussion

### Fabrication of flexible and tunable gold nanocups platforms

**Figure 1:**
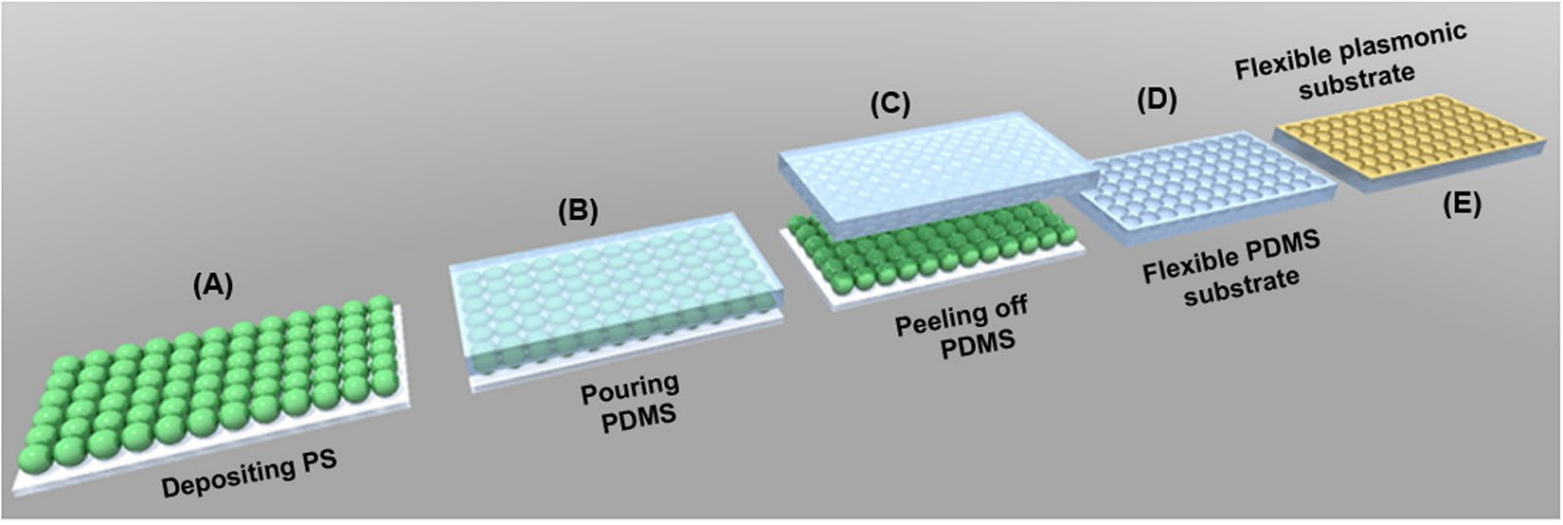
Schematic illustration of the fabrication steps of the flexible 3D Au nanocups platform. **(A)** Deposition of a PS monolayer on a glass microscope substrate by convective self-assembly method. **(B)** Pouring PDMS onto the deposited PS. **(C)** Peeling-off the PDMS film. **(D)** Obtaining the flexible PDMS substrate with impregnated nanocups. **(E)** Coating the as-prepared flexible nanocups with Au film to obtain a flexible plasmonic substrate.

The procedure employed for the fabrication of large-area flexible plasmonic nanocups platforms is described schematically in Fig. 1. Specifically, the platforms were obtained by using monodisperse polystyrene nanospheres self-assembled into a hexagonally close-packed (hcp) monolayer configuration as template for lithography and the soft lithography of the transparent polydimethylsilane (PDMS) elastomer employed as a mold for pattern replication. For that, the first step was to treat in Ultraviolet Ozone cleaning system (PSDP-UVT, Novascan) for 20 min the clean microscope slides in order to have an efficient and hydrophilic glass surface. Second, the colloidal PS of 527 nm (PS 527), 600 nm (PS 600), 719 nm (PS 719) and 836 nm (PS 836) diameter, respectively, were deposited on the as-treated microscope slides by the convective self-assembly method^26^. Then, a transparent PDMS elastomer obtained by a mixture of polymer precursor and curing agent at a 10:1 ratio was slowly poured onto the deposited PS, covering thus the PSs and filling all the voids. This hybrid substrate was cured in an oven for 1 h at 60 °C. Subsequently, the cured PDMS was carefully peeled off from the PS template and the resulting flexible substrates -denoted later as PDMS PS 527, PDMS PS 600, PDMS PS 719 and PDMS PS 836, respectively, were immersed in Dimethylformamide (DMF) to dissolve any residual PS. The as-obtained flexible nanocups array surfaces were used as a mold for the fabrication of plasmonic platforms by coating its top side surface with a 50 nm Au film using a Prevac deposition equipment (Prevac, Poland). As a result, we obtained flexible large-area with ordered Au nanocups array platforms (see Figure S1), denoted as Au PDMS PS 527, Au PDMS PS 600, Au PDMS PS 719 and Au PDMS PS 836, respectively.

### Morphological and optical characterization of the fabricated flexible Au nanocups platforms

**Figure 2:**
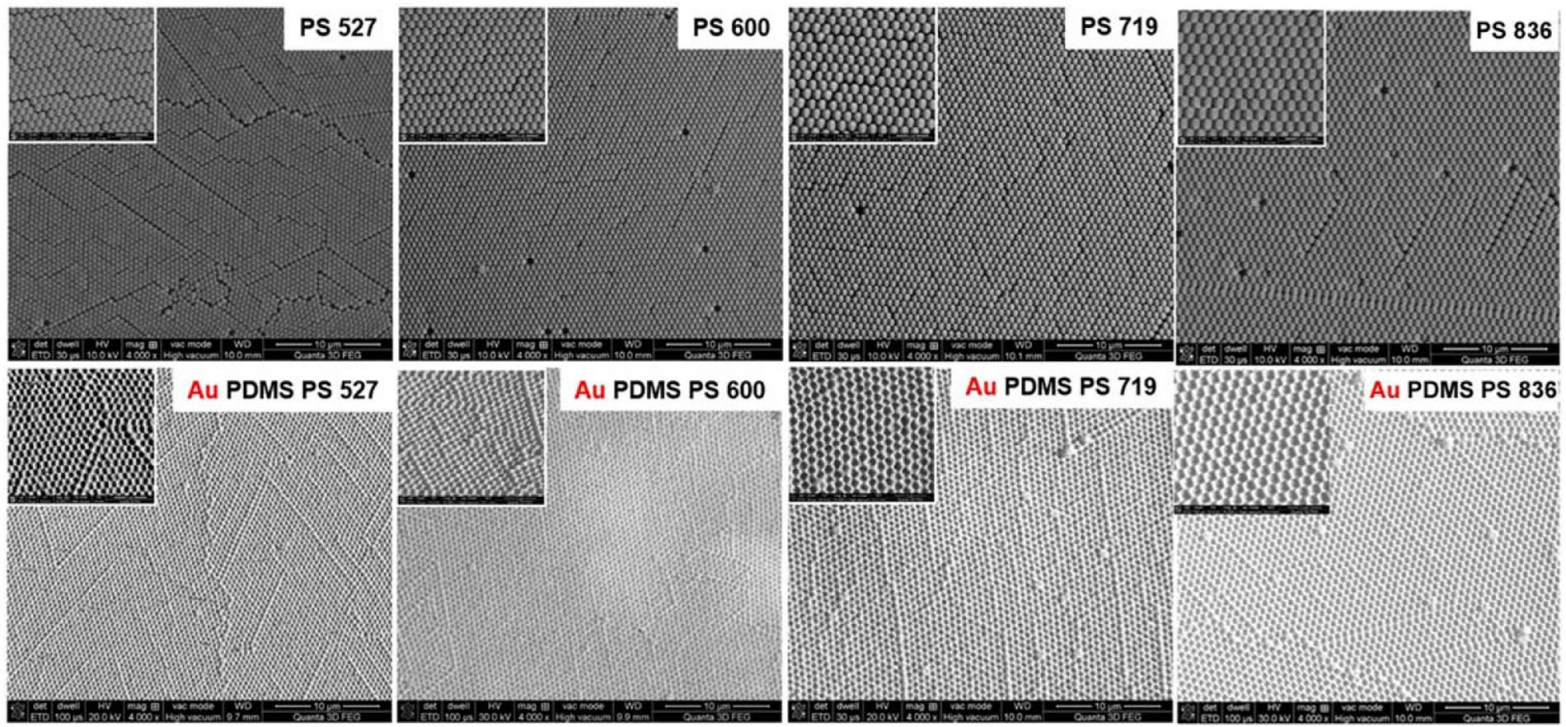
Typical SEM images at lower and higher (inset with scale bar of 2 µm) magnification of the self-assembled PS on glass substrate and the resulting PDMS film with impregnated nanocups sputtering with Au layer. (**Top**) The monolayer of the self-assembled PS with four different diameters used as templates to obtain nanocups on the flexible PDMS film. (**Bottom**) the corresponding Au-coated PDMS nanocups platforms.

Figure 2 shows representative scanning electron imaging (SEM) images of the self-assembled PS monolayer with four different diameters on glass substrates that were used as templates to obtain impregnated nanocups on the flexible PDMS film and the resulting PDMS nanocups sputtering with Au layer. The recorded SEM images show that PSs self-assembled very uniform on a large area, being closely packed on the glass substrate. Furthermore, for PS 719, the surface homogeneity is better than in the other cases, no major defect being observed. Consequently, the resulted periodically ordered arrays are perfect continuous nanocups on a large area, confirming thus the high uniformity of the fabricated nanoplatforms.

**Figure 3:**
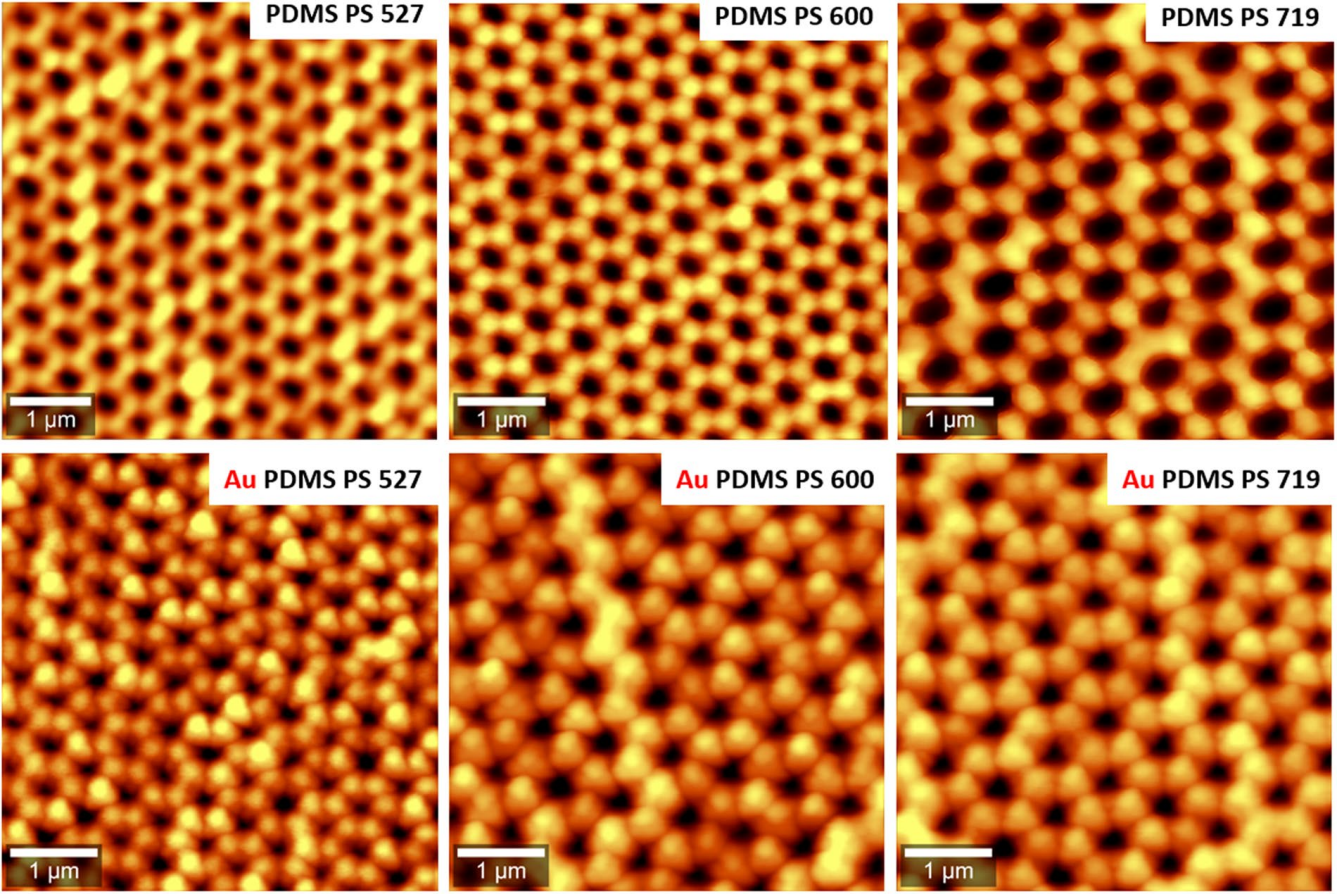
Representative AFM images of flexible nanocups platforms before and after Au deposition film. (**Top**) The flexible PDMS film with impregnated nanocups of three different diameters, (**Bottom**) Au-coated PDMS film with impregnated nanocups of three different diameters.

Subsequently, in order to illustrate the uniformity and periodicity of the fabricated flexible PDMS nanocups before and after deposition of the Au film, we have also performed atomic force microscopy (AFM) imaging. Specifically, Fig. 3 shows the recorded AFM images of the 3D flexible nanocups platforms before and after deposition Au film having three different diameters fabricated on the transparent PDMS surface. The recorded AFM images show perfect continuous nanocups arrays on a large area, the diameters of the Au-coated nanocups being smaller than the diameters of nanocups due to the presence of thickness of the metallic film. The period of the array is determined by the diameter of the PS used in each case. Additionally, in order to assess the uniformity of nanoplatforms, we have recorded a large area AFM image (20 µm × 20 µm) presented in Figure S2.

**Figure 4:**
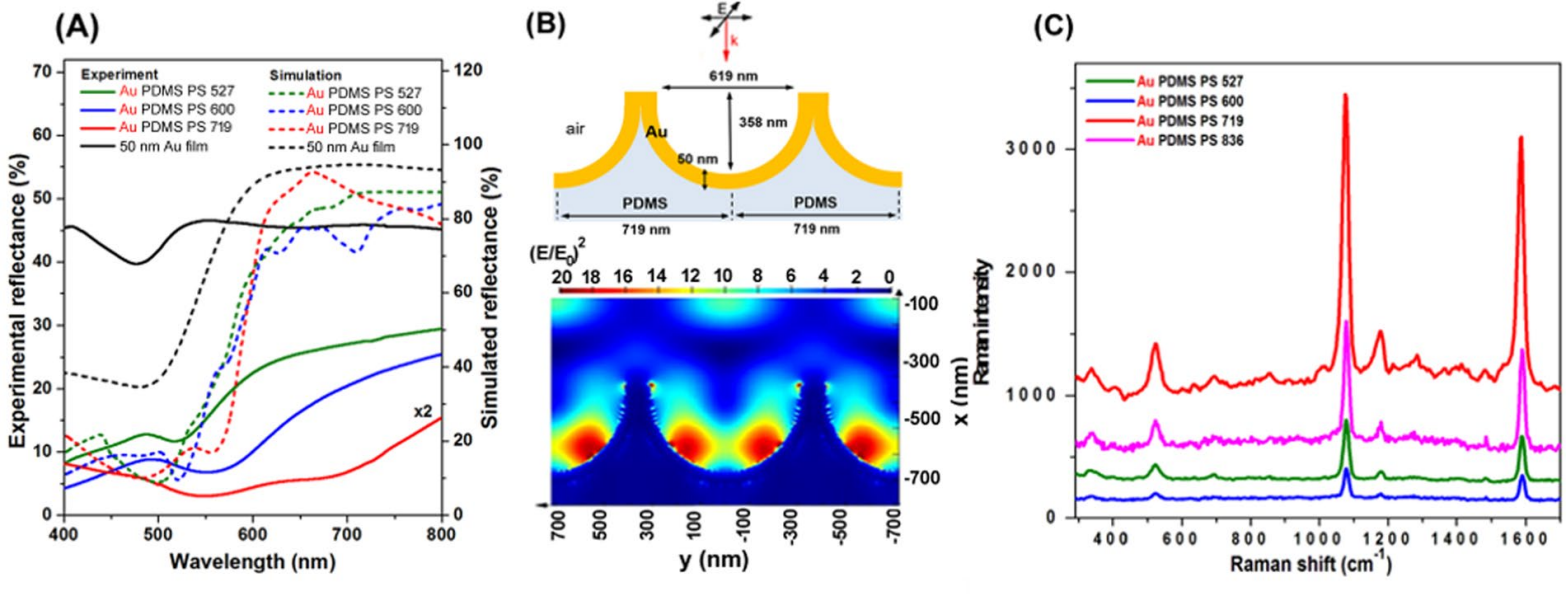
The optical response of the fabricated flexible gold nanocups platforms together with their SERS efficiency. **(A)** Measured (solid spectra) and simulated (dotted spectra) reflectance of Au PDMS PS 527, Au PDMS PS 600 and Au PDMS PS 719. **(B)** Schematic illustration of the Au PDMS PS 719 substrates considered in simulation together with the distribution of the electromagnetic field intensity (E/E_0_)^2^ for Au PDMS PS 719 at 633 nm. E_0_ is the incident light and it is equal to 1. **(C)** SERS spectra of 4-MBA adsorbed on the flexible plasmonic nanocups with different diameters: Au PDMS PS 527 (green spectrum), Au PDMS PS 600 (blue spectrum), Au PDMS PS 719 (red spectrum) and Au PDMS PS 836 (magenta spectrum) with excitation laser at 633 nm.

The experimentally measured optical reflection spectra of the obtained Au PDMS PS 527, Au PDMS 600 and Au PDMS 719 nanoplatforms are shown in Fig. 4A-solid spectra. Specifically, Fig. 4A shows that the optical responses of these 3D Au nanocups arrays platforms can be spectrally tuned by changing the diameter of the nanocups, an important feature that should be taken into consideration for optimal SERS biosensing. Bartlett *et al*. have extensively studied, both theoretically and experimentally, the plasmonic properties of Au cavity arrays^32–34^, concluding that are two dominate plasmonic modes (i.e. namely “D” or “P”). They explained that the plasmonic band appearing at longer wavelength is due to the surface plasmon generated near the center of the nanovoid, this one being called “P” mode, whereas the “D” mode confines light near the bottom of the nanovoid. However, these two optical modes were tunable with the size of the void of the plasmonic nanostructures. While a flat Au film exhibits the well-known transparency in green and high reflectivity at longer wavelengths (see Fig. 4A), in the case of our fabricated nanoplatforms, localized and propagating SPR modes are intercoupled, being difficult to estimate how much contribution comes from each mode. The dependence of the position of the transmission and reflection band on the Au hole diameter was also reported in our previous studies, the recorded plasmonic response being also explained in those cases as a coupling between LSPR and SPPs^35^. To note that the free PDMS film with impregnated nanocups (non-coated with Au film) is transparent and consequently no reflection band was recorded (data not shown).

In order to confirm our experimental results regarding the optical properties of the fabricated nanoscups platforms - Au PDMS PS 527, Au PDMS 600 and Au PDMS 719, respectively – we have performed FDTD-based numerical simulations using the commercially available FDTD Solutions software from Lumerical Inc^36^. Taking into account the experimental details, we have considered for the simulation hexagonal array units of hollow gold coated semi-spheres imprinted in PDMS (n = 1.4), having the dimensions exemplified in Figure S3. All structures were placed in air (n = 1). We have considered for gold the optical constants described by CRC data tables^37^. We used in our simulations two plane sources with wave vectors k in the x direction, normal to the plan comprising the nanostructures, and electromagnetic fields E_0_ orthogonally polarized, in the y and z directions, respectively. A 1 nm grid was employed. In order to imitate the periodicity of the array, we used periodic boundary conditions in y and z directions, while perfect matched layers (PMLs) approach was chosen on the x direction, at the top and bottom of the structure, for providing absorption boundary conditions. The local reflectivity spectra obtained for the three considered substrates are presented in Fig. 4A-dotted spectra. The reflectivity minima obtained for the considered structures, due to the excitation of surface plasmon modes, are located at 500 nm, 520 nm and 556 nm. Moreover, similar to the experimental results, the reflectivity minimum shifts to longer wavelengths with the increase of the hole diameter. In fact, the simulated spectra using theoretical sizes of nanocups are in good agreement with the experimental spectra even so in the red and near-infrared side exhibiting shallow reflectivity minima which are not exactly captured by the experimental measurements. This is because the experimental spectra are recorded from a large area (several mm square) which includes light scattered by non-patterned patches of film, line defects, film roughness etc, features which cannot be included in FDTD simulations.

### Effect of plasmonic tunability of the gold nanocups platforms on SERS performance

For a more sensitive biodetection of analytes at low concentration, the tunability of the surface plasmon resonances of the flexible nanoplatforms is an essential step to allow the maximization of the SERS enhancement^28^. For this purpose, the SERS efficiency as well as the reproducibility of the as-fabricated flexible Au nanocups platforms with different diameters were further investigated using 4-MBA as Raman probe molecule. Specifically, the SERS performances of the nanoplatforms were tested using two different excitation wavelengths that are 532 and 633 nm. As a first observation, all four nanoplatforms were found to be SERS active at both 532 (Fig. S4) and 633 nm (Fig. 4C) laser lines, characteristic bands of 4-MBA being recorded in all cases, although their SERS efficiencies are different. Kahraman *et al*. also demonstrated that the excitation laser is critical to assess the SERS performance from a nanoplatform with different diameters and depths^28^. It is evident that different degrees of enhancement of the recorded Raman signals are correlated with the reflectivity spectra presented in Fig. 4A.

In particular, the prominent vibrational bands at 1080 and 1586 cm^−1^ are assigned to ring breathing and axial ring deformation modes respectively, according to the literature^38^. The selected spectra in Fig. 4C, clearly reveal that all nanoplatforms yield high quality SERS spectra under 633 nm excitation, with Au PDMS PS 719 exhibiting the best performance in terms of sensitivity in comparison with Au PDMS PS 600, Au PDMS PS 527 and Au PDMS PS 836, respectively. This result is not surprising considering that the maximum enhancement should be achieved when the wavelength position of the LSPR of the nanostructure is between the excitation (λ_exc_) and the wavelength of Raman (λ_RS_) signal of analyte^39^, which is also our case.

Contrarily, at 532 nm only a modest SERS performance in terms of band intensities and signal to noise ratio is noticed for all three nanoplatforms (Fig. S4). The low SERS signal at 532 nm excitation is related to the well-known damping effect which arises at laser excitation line around the electronic interband transitions of gold metal^40^. Additionally, it is important to note that the reproducibly of the measurements, one of the most important parameter in the development of analytical SERS-based immunoassay, was also tested by collecting SERS spectra from several different points on the designed nanoplatforms. The intensity and the spectral position of the recorded SERS bands are constant from point to point on the nanoplatforms. Furthermore, the reproducibility of the SERS signal intensity was further quantified by averaging the values of relative standard deviation (RSD) calculated from the major SERS band located at 1080 cm^−1^ (Fig. S5). The obtained RDS values are 14,47% for Au PDMS PS 527, 17,5% for Au PDMS PS 600, 7,26% for Au PDMS PS 719 and 11,2% for Au PDMS PS 836, respectively, all these values being below 20%^41^ and indicating thus that the fabricated nanoplatforms have excellent surface uniformity, especially Au PDMS PS 719. Furthermore, in order to evaluate the limit of detection of this Au PDMS PS 719, the nanoplatform was functionalized with 4-MBA analyte at concentration between 10^−3^ M and 10^−12^ M. Figure S6 depicts the plot of the SERS peak intensities at 1080 cm^−1^ versus the 4-MBA concentration from 10^–3^ to 10^−12^ M, achieving an extended limit of detection of 10^−12^ M *via* SERS (Fig. S6A) compared with 10^–6^ M *via* LSPR measurements (Fig. S6B), proving the high sensitivity of Au PDMS PS 719 nanoplatform. To note that the SERS measurements were conducted in this case for an average of ten spots with fast acquisition time (0.1 s).

In order to get a better insight into the origin of the SERS signal, we have simulated the distribution of the field at the surface of Au PDMS PS 719 substrate at normal incidence. We considered the incident light radiating at 532 nm and 633 nm in order to match the excitation sources used in our SERS experiments. Figure S4(A) and B illustrate the relative electromagnetic field intensity ((E/E_0_)^2^) at 532 nm and 633 nm calculated along the transversal section of the nanostructure. The images indicate the presence of intense electromagnetic fields localized at corners and inside cavities pointing thus to the origin of the observed amplification of Raman signal through SERS. Moreover, the electric field intensity in these FDTD simulations is stronger at 633 nm than at 532 nm, which is in good agreement with our experimental SERS results showing that the SERS detection is improved at 633 nm excitation. This improvement obtained by Au PDMS PS 719 using a 633 nm excitation line confirms that this nanoplatform can be subsequently explored as excellent SERS biosensing nanoplatform.

### Dual label-free LSPR-SERS sensing detection on flexible gold nanocups platform

**Figure 5:**
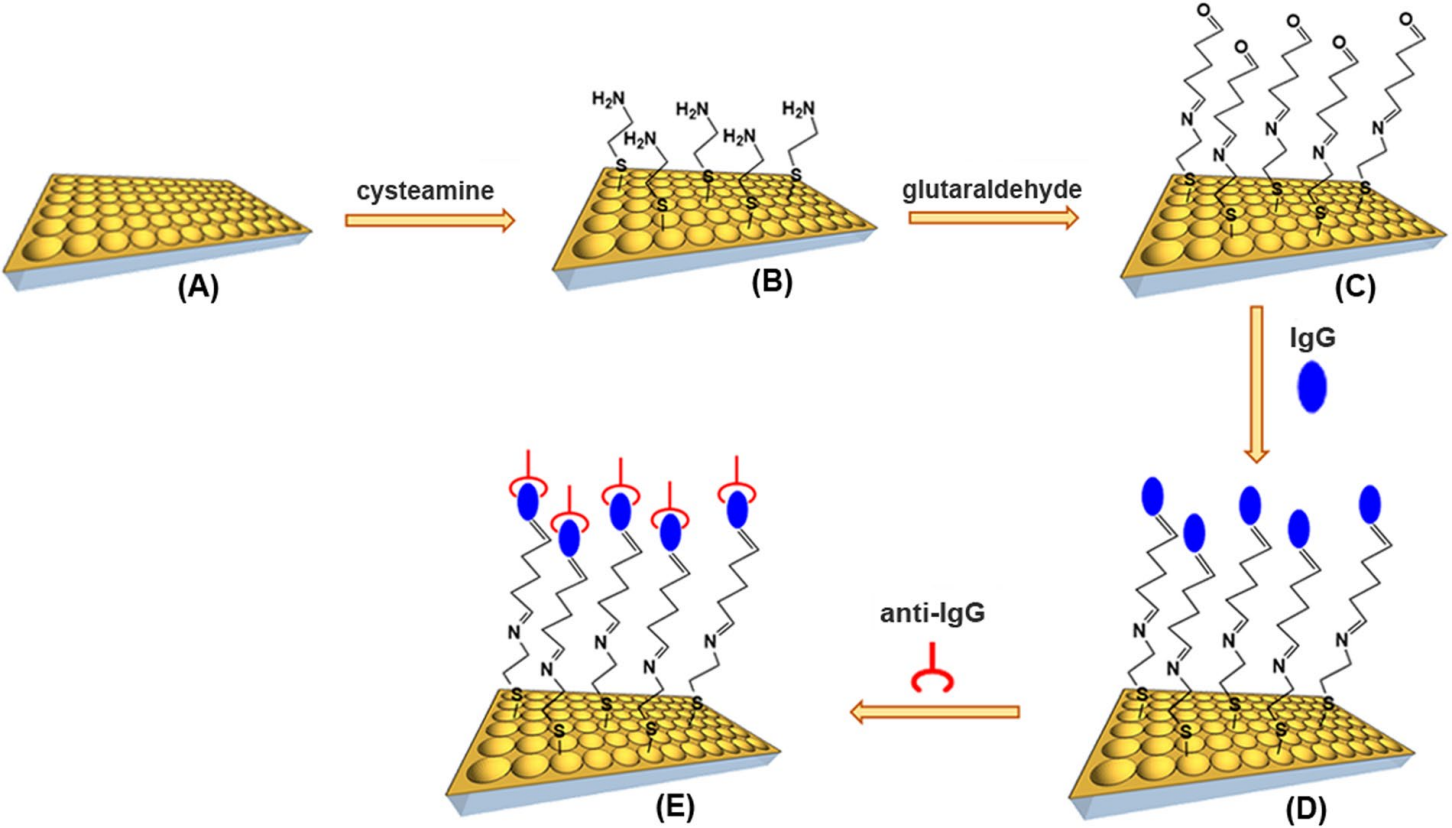
Schematic illustration representing the functionalization steps involved in the fabrication of the flexible plasmonic nanoplatform for anti-human IgG detection. (**A**) The as-fabricated flexible gold nanocups, (**B,C**) Their chemical functionalization with cysteamine and glutaraldehyde, (**D**) Human IgG immobilization, (**E**) Anti-human IgG specific detection.

As already pointed out in the introduction, we should take profit of the synergistic combination of LSPR-based nanosensing, which allows to detect the presence of analytes and SERS-based nanosensing, which specifically identifies the targeted analytes, being closer to develop the next generation of smart flexible healthcare nanosensors platform. The schematic illustration of the involved functionalization steps for the fabrication of the flexible plasmonic nanoplatform for the “proof-of-concept” direct detection of anti-human IgG in buffer serum is presented in Fig. 5.

**Figure 6:**
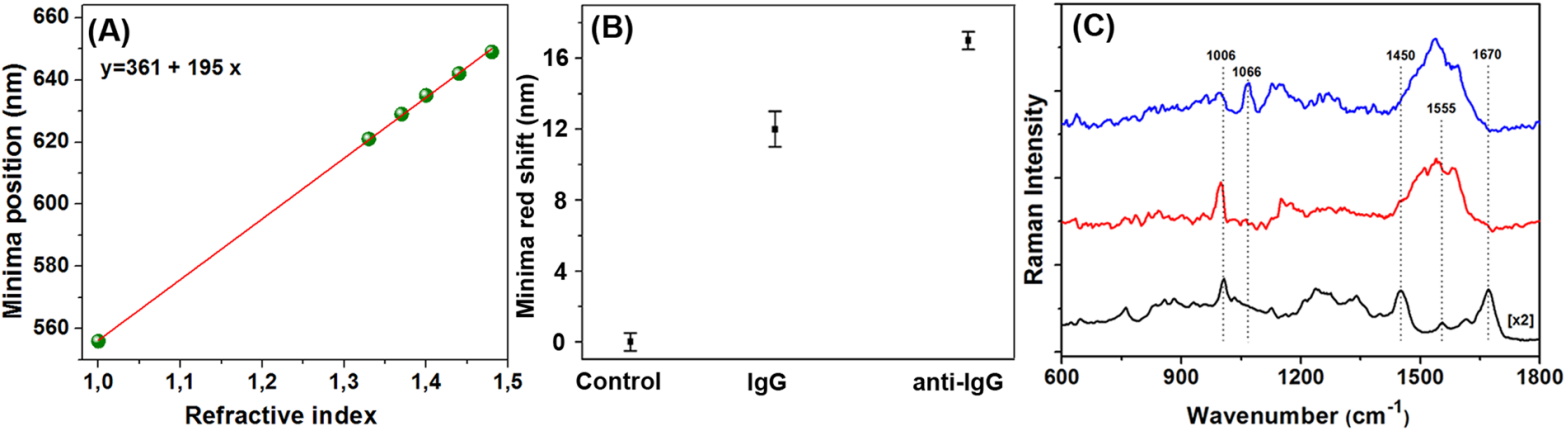
Dual LSPR-SERS specific detection of anti-human IgG. **(A)** Simulated relationship between the spectral position of reflectance spectra and the refractive index. The line is a linear fit, with the sensitivity determined to be 195 nm/RIU. **(B)** Reflectance response after human IgG immobilization and specific anti-human IgG detection. **(C)** Normal Raman spectrum of solid human IgG (black spectrum) and SERS spectra of human IgG-immobilized flexible Au PDMS PS 710 nanoplatform before (red spectrum) and after (blue spectrum) anti-human IgG attachment.

The first step was to assess the biosensing performance of the flexible Au PDMS PS 719 platform, by simulating the sensitivity of the fabricated substrate to the change of the local refractive index (RI). In general, the bulk refractive index sensitivity (S) of a LSPR nanosensor is dependent on its size, shape, and material composition^42^ and is defined as the wavelength shift of the LSPR peak in response to the change of the refractive index of the surrounding medium (expressed in nanometers (nm) per refractive index unit (RIU)). S was theoretically calculated in this case from the plot in Fig. 6A, showing the dependence of the reflectivity response position against the RI of the surrounding media for the indicated values, according to the Drude model^43^. After performing a linear regression, the slope of the fitted line indicated a bulk sensitivity of 195 nm/RIU for our Au PDMS PS 719 nanoplatform, with close S values obtained at 211 and 201 for Au PDMS PS 527 and Au PDMS PS 600, respectively (see Fig. S7). Although the designed nanoplatforms present similar bulk LSPR sensitivity, we already demonstrated that the Au PDMS PS 719 exhibits the best performance in term of SERS sensitivity and therefore this nanoplatform was selected to be applied in the detection of the specific antigen-antibody binding event.

Subsequently, the Au PDMS PS 719 flexible Au nanocups nanoplatform was employed for the “proof-of-concept” direct detection of anti-human IgG in buffer serum as a model bioanalyte, which is well-known to exhibit strong and specific binding to the heavy chains of IgG^44^. The detection of this immune reaction is important for the determination of immune system related-diseases. However, up to now different labeled-SERS immunoassays have been designed for the identification of this binding event, this strategy being preferred especially considering its higher sensitivity as compared with label-free SERS immunoassay^45^. As such, different limits of detection have been achieved and reported in literature^44,46,47^.

The reflectance minima as optical readout for anti-human IgG detection is advantageous over many other detection techniques because the detection is label-free, being easily measurable by a simple portable UV-Vis spectrometer, avoiding the necessity of expensive tools^48,49^. In our case, to check whether the anti-human IgG were successfully chemically immobilized onto the flexible Au PDMS PS 719 nanoplatform, we have performed reflectance measurements at each modification step. A reliable immobilization of human IgG antibody is fundamental for specific anti-human IgG detection and with this aim in mind we firstly immune-functionalized our sensing nanoplatform using a covalent bonding strategy well-described in the Experimental Section for IgG immobilization. As shown in Fig. 6B, the reflectance spectrum for IgG-immobilized nanoplatform is red-shifted with 12 nm compared to reflectance spectrum of Au PDMS PS 719 (referred as control in figure), due to the change of the local refractive index, indicating the successful functionalization. Subsequently, when the flexible Au PDMS PS 719 is exposed to the anti-human IgG sample, this target analyte binds to the recognition element immobilized on our nanoplatform, forming an immunocomplex. As a result, a supplementary red shift of 5 nm was recorded, which is generated by the specific detection of anti-IgG molecules.

Due to the benefit of ultrahigh sensitivity and specificity, SERS has emerged as an ideal diagnostic tool for the identification of molecular species, providing rich structural and quantitative information from their unique vibrational Raman fingerprint in a non-destructive and label-free manner^29,50^. Moreover, due to its sensitivity to the first layer of the adsorbed molecules and their orientation, SERS was proven to be a genuine technique to detect the (bio) chemical changes arising from molecular interactions. Additionally, compared to conventional biological detection techniques like mass spectrometry (MS) which offers high sensitivity but implies purification of the protein sample before analysis^51^ or the popular ELISA which requires multiple reactants and is time-consuming and expensive, SERS has the advantage over these techniques to be used as successful POC tool because of its excellent multiplexing detection capability, single biomarker sensitivity, high photostability and ease-to-use without complicated sample preparation^52–54^.

This is why, beyond LSPR measurements, label-free SERS-based immunoassay was performed further to study the specific human IgG - anti-human IgG binding event. To note that the detection of antibody-antigen recognition interactions represents the primary step in immunoassays and diagnostic applications^55^. As shown in Fig. 6C – black spectrum exhibits feature characteristic to human IgG. In particular, the Raman peaks at 1006, 1450 and 1670 cm^−1^ are assigned to the C-C symmetric stretching of phenylalanine residues, COO symmetric stretching of carboxylic acid and amide I band due to C=O stretching vibrations of the peptide moieties, respectively^56,57^. The weak band located at 1555 cm^−1^ is attributed to amide II vibration due to C–N stretching modes of the polypeptide chains. The successful adsorption of human IgG molecules onto the modified-Au PDMS PS 719 nanoplatform leads to obvious changes of Raman peak intensities and positions (Fig. 6C-red spectrum). Specifically, the vibrational Raman band at 1670 cm^−1^ disappears, while the weak peak located at 1555 cm^−1^ experiences a 15 cm^−1^ red shift together with a significant Raman enhancement. Also, for the adsorbed human IgG molecules the SERS band located at 1450 cm^−1^ significantly decreases and the band at 1006 cm^−1^ has a 7 cm^−1^ red shift. Remarkable changes of the SERS fingerprint are noticed in Fig. 6C-blue spectrum, after the specific binding of the target anti-human IgG to human IgG. In particular, the peak at 999 cm^−1^ decreased significantly, while a new band at 1066 cm^−1^ emerged. This band is connected to C–H in-plane deformation vibration of phenylalanine residues^57^. It is well known that the maximum enhancement in SERS is achieved for the vibrational modes that involve a large change of the polarizability perpendicular to the metal surface. In addition, the molecular orientation causes the shift of the corresponding vibrational bands and the change of their relative intensity. Considering the above-mentioned observations, the spectral modifications observed in Fig. 6C clearly prove the adsorption of human IgG molecules onto the Au nanocups platform and the detection of the anti-human IgG molecules using the SERS substrate functionalized with human IgG, with a detected concentration value of about 1.5 µg/mL suitable for detection in the clinical range.

## Conclusions

In summary, we introduce here a new type of flexible synergistic LSPR-SERS biosensing platforms employing uniform imprinted arrays of Au nanocups in a large area (>2 cm^2^) on a transparent and biocompatible PDMS surface. In fact, by simply changing the size of the polystyrene spheres used as templates (*i.e*. PS with diameters of 527 nm (PS 527), 600 nm (PS 600), 719 nm (PS 719) and 836 nm (PS 836), respectively) we can modulate the plasmonic response of the nanoplatforms, controlling also the enhancement of 3D electromagnetic field. Further, the bulk sensitivity of the nanoplatform with the best SERS performance (*i.e*. Au PDMS PS 719 nanoplatform) was assessed by simulating the reflectance spectra as a function of the surrounding refractive index. Then, the as-fabricated flexible Au nanocups biosensor was prepared through functionalizing the flexible nanocups array nanoplatform with human IgG as recognition element for the specific detection of the anti-human IgG and applied it to proof-of-concept direct label-free detection of anti-human IgG in buffer serum. Specifically, while the anti-human IgG detection was demonstrated by the recorded red shift of the plasmonic response of the nanocups array, the SERS technique -due to its fingerprinting abilities, proves the capture of target antigen by specific molecular identification of the human IgG - anti-human IgG recognition interaction. As such, by combining the LSPR investigation with the reliable ultrasensitive SERS technique we are able not only to confirm the presence of anti-human IgG but also to certify unambiguously the target identification after that the binding event has occurred. The development of such flexible dual LSPR-SERS-based nanosensors could be implemented for portable, inexpensive, label-free and real-time detection of relevant biomarkers with high sensitivity and specificity in the point-of-care diagnostics applications.

## Methods

### Materials

4-mercaptobenzoic acid (4-MBA), glutaraldehyde, cysteamine, Bovine Serum Albumin (BSA, 66 kDa), Immunoglobulin from human serum (human IgG) and anti-human IgG were purchased from Sigma-Aldrich. Poly(dimethylsiloxane) (PDMS) prepolymer and a curing agent were obtained from Dow Corning (Sylgard 182, Midland, MI, USA). Monodisperse polystyrene nanospheres (PS) suspensions (2.5 or 5 wt % in water, surfactant free) with diameters of 527, 600, 719 and 836 nm, respectively, were bought from MicroParticles. All chemicals were used as received.

### Testing SERS efficiency

To probe the SERS efficiency of the as-obtained flexible Au nanocups array platforms, 4-MBA was selected as a covalently binding Raman probe molecule. In particular, the Au PDMS PS 527, Au PDMS PS 600, Au PDMS PS 719 and Au PDMS PS 836  platforms were first immersed in a 10^−6^ M 4-MBA methanolic solution for 60 min, and then extensively rinsed to ensure that only one molecular layer was adsorbed on the flexible plasmonic substrate.

### Biosensing protocol

The biosensing protocol proposed for the detection of anti-human IgG antibody is schematic illustrated in Fig. 5. The SERS optimized flexible nanoplatform (herein Au PDMS PS 719) was first immersed in 0.2 M cysteamine aqueous solution in darkness at room temperature for 15 h to form a cysteamine monolayer on the gold nanocups substrate. The substrate was then thoroughly washed in ultrapure water to remove physically adsorbed cysteamine. Subsequently, 4% glutaraldehyde with two aldehyde functional groups reacted with cysteamine modified-substrate at room temperature for 4 h, by forming a new imine bonding between cysteamine and glutaraldehyde molecules. Next, the substrate was thoroughly washed with PBS buffer (pH 7.4) to remove the physically adsorbed glutaraldehyde. To immobilize the capture human IgG on the aldehyde-terminated surface, the transducer platform was incubated in PBS (pH 7.4) containing 1 mg/mL human IgG at 20 °C for 15 h and rinsed then with PBS buffer. Nonspecific binding was prevented by using 5 mg/mL of BSA in PBS for 1 h. This step was immediately followed by 4 h incubation with 50 μL of anti-human IgG (1.5 µg/mL).

### Characterization methods

The morphology of the flexible PDMS film with impregnated nanocups and Au-coated PDMS film with impregnated nanocups of three different diameters was analyzed using a Witec Alpha 300 A atomic force microscope (AFM) operated in tapping mode. The AFM image was recorded on an area of 5 µm × 5 µm, 256 × 256 pixels at a moving speed of 1 s per line. The self-assembled PS on glass substrate with four different diameters and the obtaining flexible Au-coated nanocups array platform on large area substrate was characterized by a FEI Quanta 3D FEG dual beam electron microscope operating at an accelerating voltage of 10 kV. The Scanning Electron Microscopy (SEM) images were recorded using an FEI Quanta 3D FEG scanning electron microscope operating at an accelerating voltage of 05–30 kV. The self-assembled PS monolayer were previously covered with a 5 nm Au layer to amplify the secondary electron signal. The LSPR response of the flexible Au nanocups arrays was recorded using a UV-Vis spectrophotometer (Jasco V 530 with SLM − 468 S reflectivity module). The SERS efficiency of the four types of nanoplatforms was tested using a Confocal Raman Microscope (alpha 300 R from WITec GmbH, Ulm, Germany) with excitation wavelength at 532 and 633 nm, respectively. The SERS signals were collected through a confocal pinhole of 100 µm by using a 0.9 NA objective of 100 × magnification. The typical exposure time was 10 s and the laser power was 1 mW at the objective exit. For investigating the SERS reproducibility and uniformity of our nanoplatforms, the measurements were performed in different arbitrarily chosen regions on the nanoplatorm. To investigate the human IgG - anti-human IgG binding events, the Raman and SERS spectra were acquired with the same alpha 300 R WITec microscope employing in this case for excitation the 632 nm wavelength from a He-Ne laser. The reference Raman spectra of human IgG were recorded through a 20 × objective (NA = 0.4). The measurements were conducted at a laser power incident on the sample of 3 mW, and the integration time was set at 3 s per spectrum.

## Electronic supplementary material

Supplementary Information
