# Supplementary material for: Flexible and Tunable 3D Gold Nanocups Platform as Plasmonic Biosensor for Specific Dual LSPR-SERS Immuno-Detection
Source: Sci Rep. 2017 Oct 27;7:14240. doi: 10.1038/s41598-017-14694-1 (PMC5660151; doi:10.1038/s41598-017-14694-1)
Supplement: Supplementary file 1 — Supplementary Information [file 41598_2017_14694_MOESM1_ESM.pdf]

Supplementary Data Available

Flexible and Tunable 3D Gold Nanocups Platform as Plasmonic Biosensor for Specific Dual LSPR-SERS Immuno-Detection

M. Focsan[a *], A. M. Craciun[a], M. Potara[a], C. Leordean[a], A. Vulpoi[b], D. Maniu[a,c] and S. Astilean[a,c*]

[a] Interdisciplinary Research Institute on Bio-Nano-Sciences, Babes-Bolyai University, Treboniu Laurean Str. 42, Cluj-Napoca 400271, Romania

[b] Nanostructured Materials and Bio-Nano-Interfaces Center, Interdisciplinary Research Institute on Bio-Nano-Sciences, Babes-Bolyai University, Treboniu Laurian Str. 42, Cluj-Napoca 400271, Romania

[c] Biomolecular Physics Department, Faculty of Physics, Babes-Bolyai University, M Kogalniceanu Str. 1, Cluj-Napoca 400084, Romania

*Corresponding author: Tel: +40 264 405300/5188; Fax: +40 264 591906; e-mail addresses: simion.astilean@phys.ubbcluj.ro (Prof Dr Simion Astilean), monica.iosin@phys.ubbcluj.ro (Dr Monica Focsan)

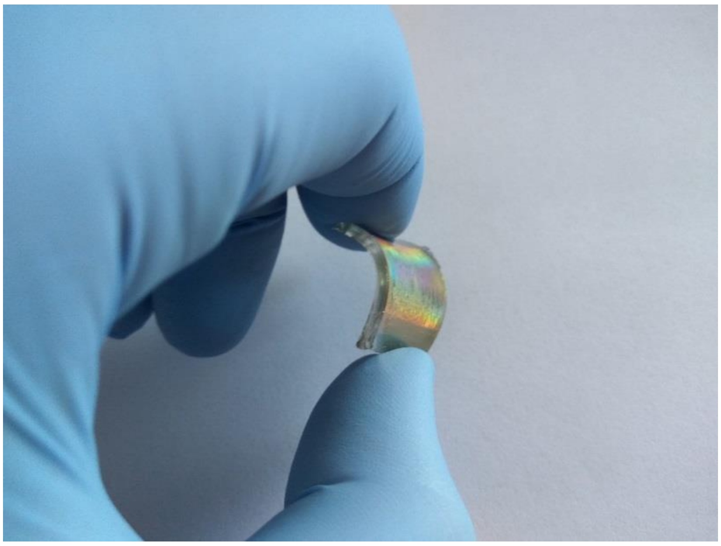

Figure S1. Digital image of the fabricated flexible Au nanocups platform.

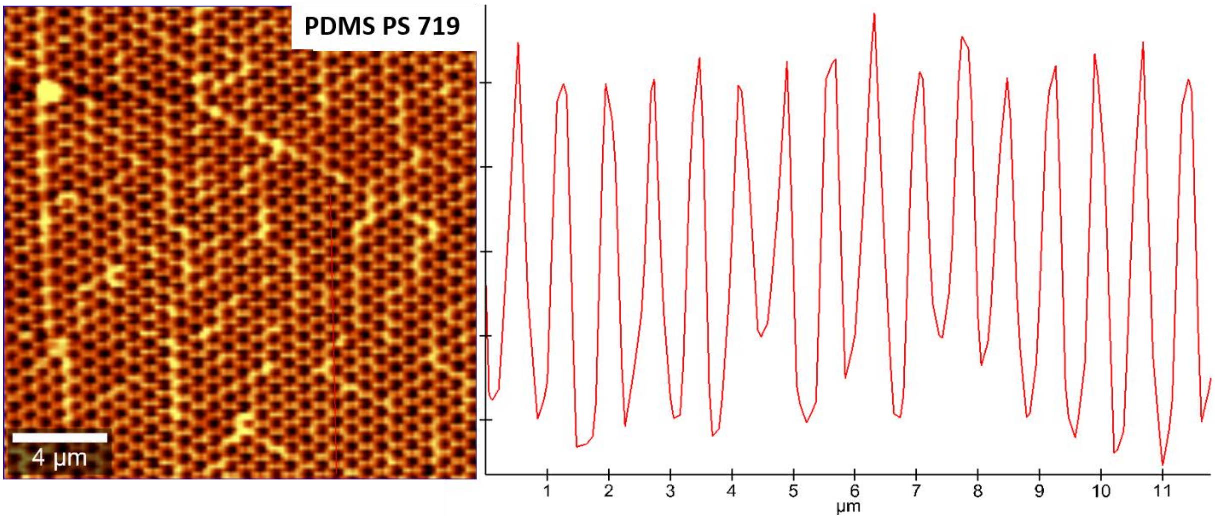

Figure S2. Representative large area AFM image of the flexible PDMS film with impregnated nanocups of 719 nm diameter together with the cross section recorded across the solid line in the AFM image.

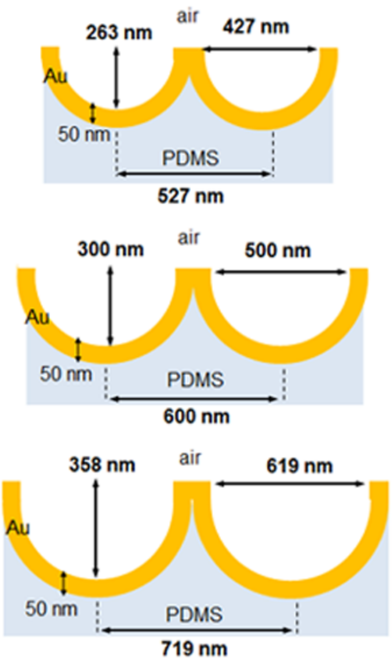

Figure S3. Schematic illustration of the nanoplatforms considered in simulation: Au PDMS PS 527, Au PDMS PS 600 and Au PDMS PS 719, respectively (from top to bottom).

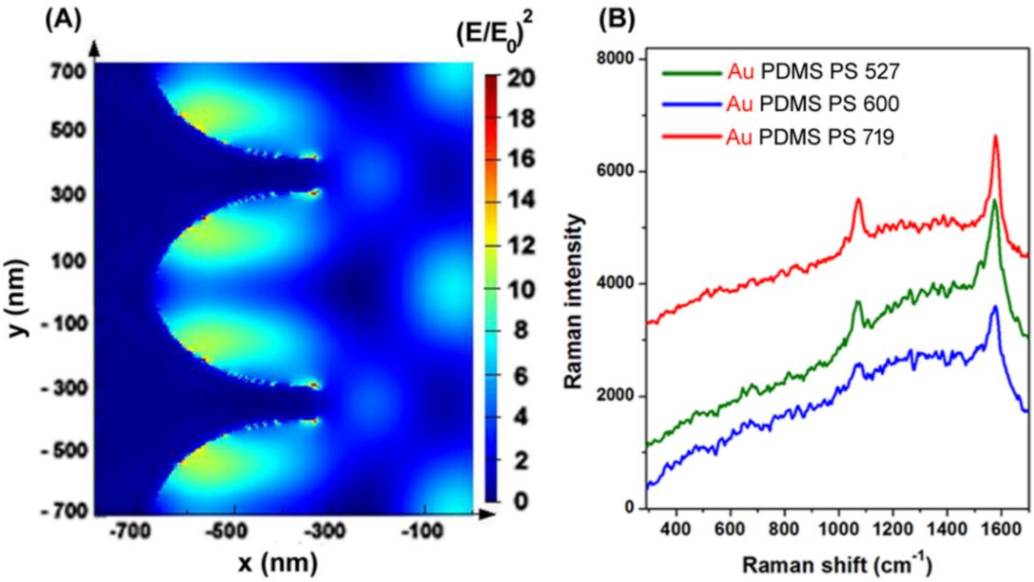

Figure S4. (A) Distribution of the electromagnetic field intensity $(E/E_0)^2$ for selected PS 719 at 532 nm. E_0 is the incident light and it is equal to 1. (B) SERS spectra of 4-MBA adsorbed on the flexible plasmonic nanocups with different diameters: Au PDMS PS 527 (green spectrum), Au PDMS PS 600 (blue spectrum), and Au PDMS PS 719 (red spectrum) with excitation laser at 532.

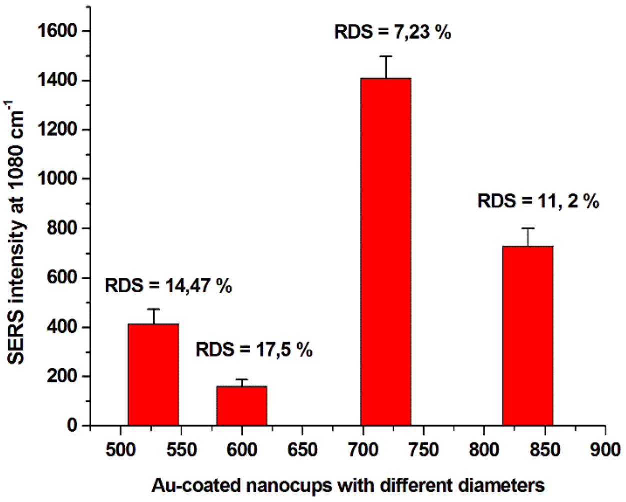

Figure S5. Reproducibility of the SERS measurements in order to validate the uniformity of the flexible Au-nanocups platforms. The intensity of the main Raman vibration of 4-MBA at 1080 cm^{-1} collected from different points from each nanoplatform and their corresponding RSD values.

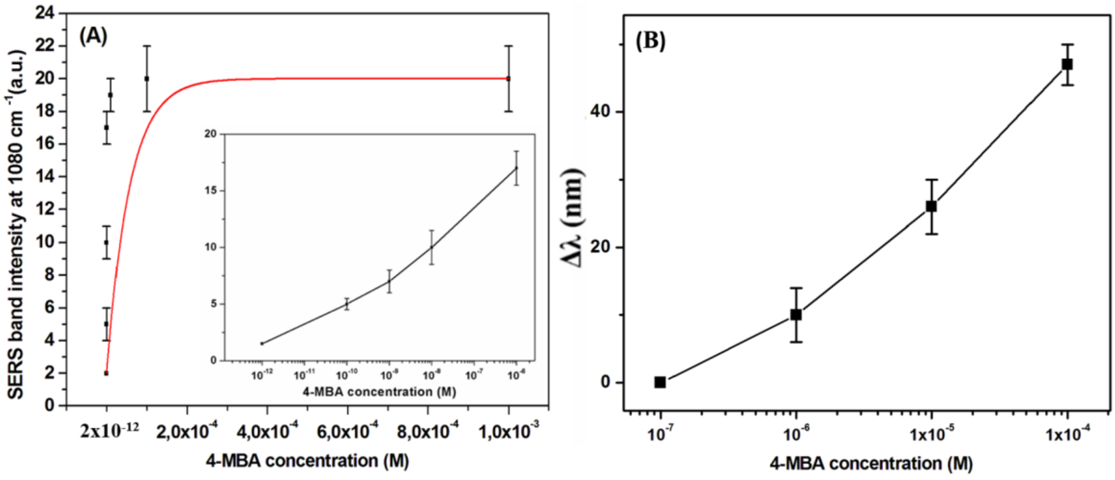

Figure S6. Evaluation of the limit of detection (LOD) of Au PDMS PS 719 nanoplatform. (A) LOD determined *via* SERS by examination of the SERS intensity response of different concentrations between 10^{-3} and 10^{-12} M of 4-MBA on Au PDMS PS 719 at 1080 cm $^{-1}$ band. The inset shows the plot in the concentration ranges of 10 $^{-12}$ to 10 $^{-6}$ M. The laser line is 633 nm. (B) LOD determined *via* LSPR by measuring the reflectance spectrum after functionalization with 4-MBA analyte at same concentrations.

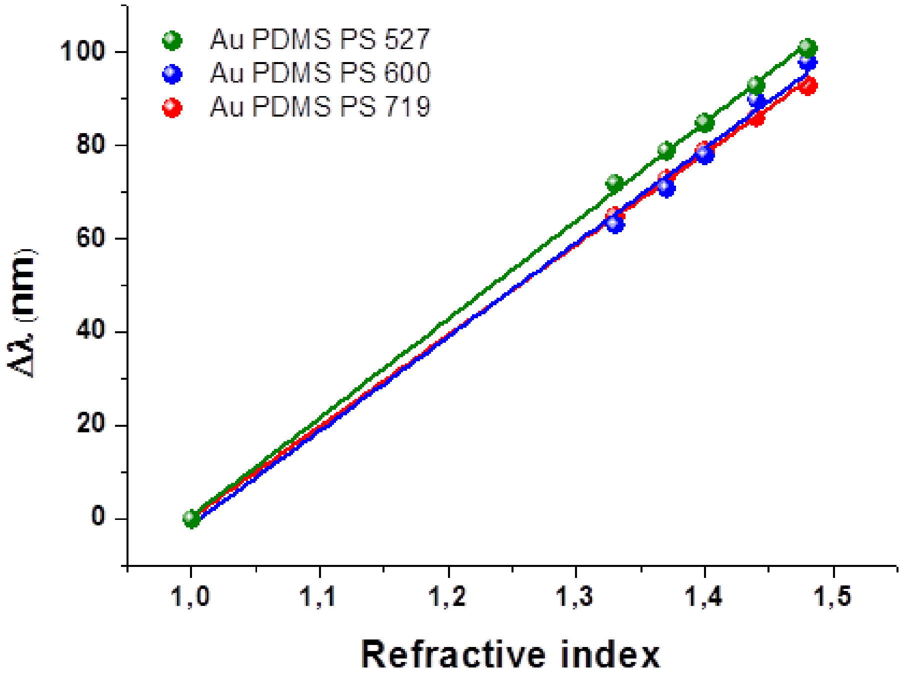

Figure S7. Simulated relationship between the spectral position of reflectance spectra and the refractive index for Au PDMS PS 527, Au PDMS PS 600 and Au PDMS PS 719 nanoplatforms. The lines are linear fit, with the sensitivity determined to be 211, 201 and 195 nm/RIU.
